# Area-based determinants of outreach vaccination for reaching vulnerable populations: A cross-sectional study in Pakistan

**DOI:** 10.1371/journal.pgph.0001703

**Published:** 2023-09-27

**Authors:** Xiaoting Chen, Allan Porter, Nabeel Abdur Rehman, Shaun K. Morris, Umar Saif, Rumi Chunara

**Affiliations:** 1 Department of Biostatistics, New York University, New York, New York, United States of America; 2 Department of Computer Science Engineering, New York University, Brooklyn, New York, United States of America; 3 Division of Infectious Diseases and Centre for Global Child Health, The Hospital for Sick Children, Toronto, Canada; 4 Dalla Lana School of Public Health, University of Toronto, Toronto, Canada; 5 Department of Paediatrics, University of Toronto, Toronto, Canada; 6 UNESCO Chair for ICTD, Lahore, Pakistan; Banaras Hindu University, INDIA

## Abstract

The objective of this study is to gain a comparative understanding of spatial determinants for outreach and clinic vaccination, which is critical for operationalizing efforts and breaking down structural biases; particularly relevant in countries where resources are low, and sub-region variance is high. Leveraging a massive effort to digitize public system reporting by Lady and Community Health Workers (CHWs) with geo-located data on over 4 million public-sector vaccinations from September 2017 through 2019, understanding health service operations in relation to vulnerable spatial determinants were made feasible. Location and type of vaccinations (clinic or outreach) were compared to regional spatial attributes where they were performed. Important spatial attributes were assessed using three modeling approaches (ridge regression, gradient boosting, and a generalized additive model). Consistent predictors for outreach, clinic, and proportion of third dose pentavalent vaccinations by region were identified. Of all Penta-3 vaccination records, 86.3% were performed by outreach efforts. At the tehsil level (fourth-order administrative unit), controlling for child population, population density, proportion of population in urban areas, distance to cities, average maternal education, and other relevant factors, increased poverty was significantly associated with more in-clinic vaccinations (β = 0.077), and lower proportion of outreach vaccinations by region (β = -0.083). Analyses at the union council level (fifth-administrative unit) showed consistent results for the differential importance of poverty for outreach versus clinic vaccination. Relevant predictors for each type of vaccination (outreach vs. in-clinic) show how design of outreach vaccination can effectively augment vaccination efforts beyond healthcare services through clinics. As Pakistan is third among countries with the most unvaccinated and under-vaccinated children, understanding barriers and factors associated with vaccination can be demonstrative for other national and sub-national regions facing challenges and also inform guidelines on supporting CHWs in health systems.

## Introduction

Immunization has been the most cost-effective measure for reducing childhood mortality and morbidity worldwide [1]. Immunization also plays a critical role in achieving 14 of the 17 Sustainable Development Goals [2]. Despite efforts to strengthen infrastructure and supply chains over the past decades, targets set by the World Health Organization’s Expanded Programme on Immunization, immunization against diphtheria, pertussis, tetanus, poliomyelitis, measles and tuberculosis for every child in the world have been a challenge in certain places. In particular, progress in several places has plateaued because of specific populations which remain hard to reach [3]. For example, vaccination rate stagnation in parts of South Asia and Africa are well-documented, with children in remote and rural villages represent a disproportionate number of non-immunized children [4–7]. Indeed, such disparities are particularly challenging in countries in which resources are low and sub-region variance is high, making it difficult to understand what works for vaccination in such diverse and vulnerable places [8, 9].

Reviews of why children remain unvaccinated have illustrated both systemic (e.g., immunization system access, health worker training and geographic/area-based) and individual level (e.g., parental attitudes and knowledge) barriers, with immunization system issues including access challenges being the most frequently cited [10–12]. Though researchers have studied multiple ways to improve vaccination rates, such as through design of immunization cards and center based education [13], immunization process improvements at the systematic level, such as improving outreach services, should be an important focus for advancement of vaccination rates [11, 14]. Accordingly, bringing immunization closer to communities is an important need.

Lady Health Workers and Community Health Workers (CHWs) [15] are known to help facilitate vaccination through a variety of means including: improving motivation to bring children to clinics and consent for immunization [16–19] and vaccination delivery via their knowledge of local areas [20, 21]. Indeed, evidence on select factors related to CHW-facilitated vaccination including increased vaccination coverage, trust and lower cost have been demonstrated in studies across many regions including India, Ghana, Mexico, Papa New Guinea, South Africa, Mozambique, Ecuador and Palestine [3, 8]. Through this research it is envisioned that CHWs will be better able to reach vulnerable populations and promote equity in the provision of primary care services [3]. At the same time, geographic barriers are an important consideration for healthcare access, including healthcare through CHWs. A recent study from Madagascar suggests that, given the same propensity to seek care in the population, decreasing geographic barriers alone or even additional CHW sites will not result in large gains in consultation rates [22]. It should be noted that this study examines CHW engagement in isolation, and does not include any comparison of CHWs and clinics. In addition to these cited gaps, studies that utilize reports of CHW use from national or individual-level surveys suffer from recall biases. On the other hand, digitalization of programs is a potentially promising avenue towards improving immunization access, by making tools portable to facilitating their use [23] as well as improving the ability to gather information on the operation of the service, a key part of health service coverage and evaluation frameworks [24]. Specifically, data can help inform a systematic understanding of the reach of outreach and clinic vaccination to vulnerable populations, for which there is currently a lack of evidence. In sum, the extant research motivates the need for further research regarding the optimal use of CHWs to improve immunization coverage. This type of analysis, especially at sub-national levels, is needed in order to focus system-level improvements. Such results are particularly important in Pakistan which is home to one of the largest populations of un- and under-vaccinated children in the world. Globally, Pakistan has the third highest burden of child mortality [25].

In this study we study the following research questions: we assess the relevance of regional spatial attributes to outreach vaccination coverage rates, clinic vaccination coverage and the ratio of outreach to all vaccinations, in province in Pakistan. Recently, a provincial-level government (Punjab) enacted a massive effort to digitize public system reporting which enabled this detailed analysis. This large, comprehensive dataset on geo-located vaccination records from an entire province of Pakistan enables study of the research questions at multiple spatial scales relevant to variations in regional attributes and the factors related to vaccination in clinics and by CHW outreach vaccination. We focus on regional area-based spatial attributes in order to address systematic barriers as motivated above. Accordingly, this study adds to the existing literature, particularly regarding comparative barriers to clinic versus outreach vaccination. Importantly, area-based factors can directly influence operations and policy. Results of the study illustrate how the regional factors related to the receipt of outreach services can be used to improve CHW operations, and to decrease barriers to basic healthcare services for vulnerable populations.

## Methods

This is a cross-sectional study, assessing the importance of regional factors in relation to Pentavalent dose 3 (Penta-3) vaccination rates in-clinic and via outreach coverage in Punjab province, Pakistan from September 2017 through December 2019. Three modelling approaches are used to distill the significance of 19 spatial factors in relation to 1) regional clinic vaccination rates, 2) regional outreach vaccination rates and 3) the proportion of all vaccination happening via outreach. Three statistical modeling methods are chosen in order to leverage different levels of flexibility to handle complex spatial data, and model interpretability, a common tradeoff in statistical learning [26]. The methods used are the Gradient Boosting Model (GBM), the Generalized Additive Model (GAM), and Ridge Regression. The vaccination data was collected by the Punjab Information Technology Board as part of public health surveillance activities and was provided fully anonymized. Details on the data and methods follow.

### Setting and information systems

Modern technology was applied by the Punjab Information Technology Board (PITB) to track containment activities carried out by the Punjab Health Department. Mobile phones were distributed to health care workers and clinics to record activities including vaccinations using a mobile application. Government workers were asked to take a picture before and after performing the activity as a verifiable proof that the activity had been performed. Global positioning system (GPS) coordinates of the location, time stamp, and pictures of the child were automatically submitted to a centralized server where they are monitored. For more detail, a similar system by the PITB has previously been described [27].

Data on vaccination activities for the period September 1, 2017, to December 31, 2019, was used. Partial data for January to April 2018 and all data for December 2019 were missing. Clinic vaccinations were not included in months 1–7 of 2017 as they were not officially being recorded as part of the same system at that time. We used the data to perform analyses to identify the relationship between multiple area-level features and Penta-3 outreach or clinic vaccinations, as described below. Third penta-3 dose was used as it indicates the complete series, prior to boosters.

### Region of analyses

Analyses were performed at the finest spatial resolution in which all relevant features were available (tehsil level). Tehsils are the fourth-order administrative divisions of Pakistan, below districts, provinces, and divisions. To assess if spatial heterogeneities were being masked, we also did a sub-analysis with available predictors at the fifth-order level (union council). There are 137 Tehsils in Punjab and five were excluded from modeling due to them not being individually accessible for many covariates (De-Excluded Area Rajanpur, De-Excluded Area D.G. Khan, Karor Lal Esan, Faisalabad Saddar, Sahiwal). For more localized analysis, there are 3445 Union Councils for Punjab. Of those, 2742 were finally included due to regions with unavailable covariates unavailable being excluded. Each vaccination was either performed in a fixed healthcare setting (clinic), or an outreach visit by a healthcare field worker (outreach), and the determination of clinic or outreach type of each vaccination record was based on whether the record’s GPS coordinates were close to any medical facilities on list (see S1 File). Third dose was used as it indicates the complete series, prior to boosters. An overall summary of number of vaccination records available by year is in Table 1.

**Table 1:** Counts of pentavalent dose 3 vaccinations administered from September 2017 through 2019 by clinic or outreach status as well as the yearly proportion of outreach vaccinations.

	In Clinic	Outreach	Outreach Proportion
**2017 (Sept.–Dec.)**	40,770	463,272	91.9%
**2018**	209,652	1,309,116	86.2%
**2019**	211,727	1,135,255	84.3%
**Total**	462,149	2,907,643	86.3%

### Area-based predictors

Area-based predictors (attributes of the local region) were included based on previous studies of childhood vaccinations of pentavalent (pentavalent vaccine protects against Haemophilus influenza B, diphtheria, pertussis, tetanus and hepatitis B) and DPT (diphtheria, tetanus, pertussis) across Africa and Pakistan [28, 29] and availability of data. In total, 19 predictors were selected for the tehsil level model, spanning concepts including area socioeconomics (percentage of people living in poverty, average households with radio, mobile phone, television, etc.), area demographics (child population, average mothers age, etc.), and healthcare resources and availability (average distance to cities in the region, urban rural ratio, number of public clinics offering vaccination, etc.). Area-based demographics and healthcare resources can shape vaccination rates based on demand and access. Moreover, a substantial amount of research has also shown that area-based socioeconomic factors, are also associated with a variety of health outcomes, independent of individual characteristics. Thus area-based attributes have been referred to as “emergent characteristics” themselves, that can predict the ability of residents to obtain health care [30]. Theoretically, this characteristic is thought to be linked to vaccination rates via multiple mechanisms. First, living in disadvantaged neighborhoods is less conducive to health. For example, if a neighborhood has poor roads or public transit, traveling to obtain needed health care may be inconvenient and costly. Second, concentrated socioeconomic disadvantage may also diminish institutional resources such as churches and schools which act as nodes in social networks through which information and social support, including that related to obtaining health care, may be obtained [31]. At the union council (UC) level, 7 out of 19 features were accessible and selected for the higher granularity analysis. Detailed information on predictors and data sources are listed in S1 Table.

All attributes were first normalized. Pearson cross-correlation was computed for the entire set of variables and outcomes to understand bi-variable relationships. For multi-variable analysis, after selection via background literature, we used feature selection procedures to assess potential relevance of each included feature to the study outcomes. Two feature selection algorithms were used: recursive feature elimination (RFE) and Boruta (detailed methods reported in S2 File). Covariates identified as important by both RFE and labelled as “confirmed” or “tentative” via Boruta were included in the next steps. Considering the tradeoffs between algorithm flexibility and interpretability (details in S4 File), we examined the importance of selected variables using the three modeling approaches (ridge regression, generalized additive model and gradient boosted machines).

### Outcomes

Three outcomes were considered. First, clinic vaccination coverage per tehsil which was computed by dividing the sum of penta-3 vaccinations administered in clinics per tehsil by their respective child populations. Second, outreach vaccination per tehsil which was computed by dividing the sum of penta-3 vaccinations administered by outreach vaccinations per tehsil by their respective child populations. These outcomes enable identification of which attributes are more relevant for outreach vaccination (positive relevance) and clinic vaccination (negative relevance) by tehsil. Finally, to distinguish which regional attributes were related to a higher proportion of vaccines being administered through outreach than clinics, we used the same modelling approaches to predict the ratio of outreach to all (outreach and clinic) vaccinations by tehsil.

### Model implementation

Three machine learning algorithms were used to evaluate the contribution of selected area-based features. The first algorithm was a Gradient Boosting Model (GBM). This model combines and improves upon simple statistical learning models by iterative corrections. In the GDM, the loss between observed and predicted values are ultimately minimized by assigning different weights on each simple model to get the best combination as the final strong predictive model. The most used simple model is decision tree, which is also the case in this analysis. GBM can capture nonlinear relationships and deal with multicollinearity and heteroskedasticity issues based on its iterative correction of errors and learning from diverse simple models, effectively modeling complex data patterns and minimizing the impact of correlated predictors and varying error structures. The second algorithm used was a Generalized Additive Model (GAM), which is an extension of generalized linear models that allows for non-linear relationships between predictors and an outcome. GAM works by decomposing the relationship between independent and dependent variables into additive components, where each component is represented by a smooth function that can capture the non-linear patterns in the data. This allows GAMs to model complex relationships while still providing interpretable results through the additive structure. The third algorithm was Ridge Regression, a linear regression model that incorporates regularization with a squared penalty term to enhance model robustness. The linear regression form of the Ridge algorithm provides clear and interpretable coefficients (in the same manner as linear regression), enabling straightforward interpretation of the magnitude and direction of the predictors’ impact on the independent variable. This model differs from other regression models by the addition the regularization term, which penalizes the model for larger coefficient values.

By adding this penalty term, ridge regression shrinks the coefficients of correlated predictor variables towards each other, reducing their individual impact. This helps in reducing the multicollinearity problem and makes the model more stable and reliable. Thus, the regularization effectively addresses multicollinearity issues, reducing the impact of correlated predictors and improving the model’s stability. Readers are referred to foundational publications for further details [26]. As has been detailed in the referenced foundational text, restrictions that decrease flexibility also improve interpretability. Thus, considering the trade-offs between algorithm flexibility and interpretability, we chose three models that offer a range of flexibility and interpretability, to together examine and compare their reported importance of selected variables. Statistical checks were conducted to ensure that the assumptions for each algorithm were met before implementing the models. Details of the model choice discussion and statistical checks are provided in the S4 File. For each of the three different modelling approaches different indicators were used to assess feature relevance: relative influence for GBM; the -log of the p-value for the smoothing function for a predictor (*s(●)*) for GAM; values of the standardized beta coefficients for ridge regression. As a smaller number of predictors were available at the union council spatial resolution, and the beta coefficients in ridge regression also provide direction (negative or positive), for interpretability we focused on the ridge models for the UC analysis (S3 File).

For evaluation of all models, training and test samples were generated using random 80/20 splits. The root-mean squared error (RMSE), R-Squared, and mean absolute error (MAE) performance, common evaluation statistics, were calculated on each test set (S2 File). For all three modelling approaches, features identified as significant in the feature selection process were included in the model fitting. Observations with missing values were dropped before model implementation. For GBMs, gbm.step function from the dismo package in R was utilized on training data in order to establish an optimal number of boosting trees to be used for this model via 10-fold cross validation. GAMs were fitted using the gam function from the mgcv package in R with smoothing parameter estimation method “REML”. Ridge models were implemented using the cv.glmnet function in the glmnet package with alpha = 0, and the hyperparameter lambda was tuned via 10-fold cross validation. Final model results are reported using the average value given by 1000 iterations of bootstrapping resampling and model fitting with estimated standard error. All models were implemented in RStudio (R version 4.2.0). Code for all analyses is located at: https://github.com/ChunaraLab/PK_Vaccination.

## Results

### Outreach vaccination efforts

From September 2017 through December 2019, of all vaccination records, 86.3% were performed by outreach efforts in Punjab Province (Table 1), demonstrating the utility of this modality of delivering healthcare services and increasing vaccination rates beyond the capacity of healthcare clinics.

### Regional attribute relevance to vaccination

**Fig 1:**
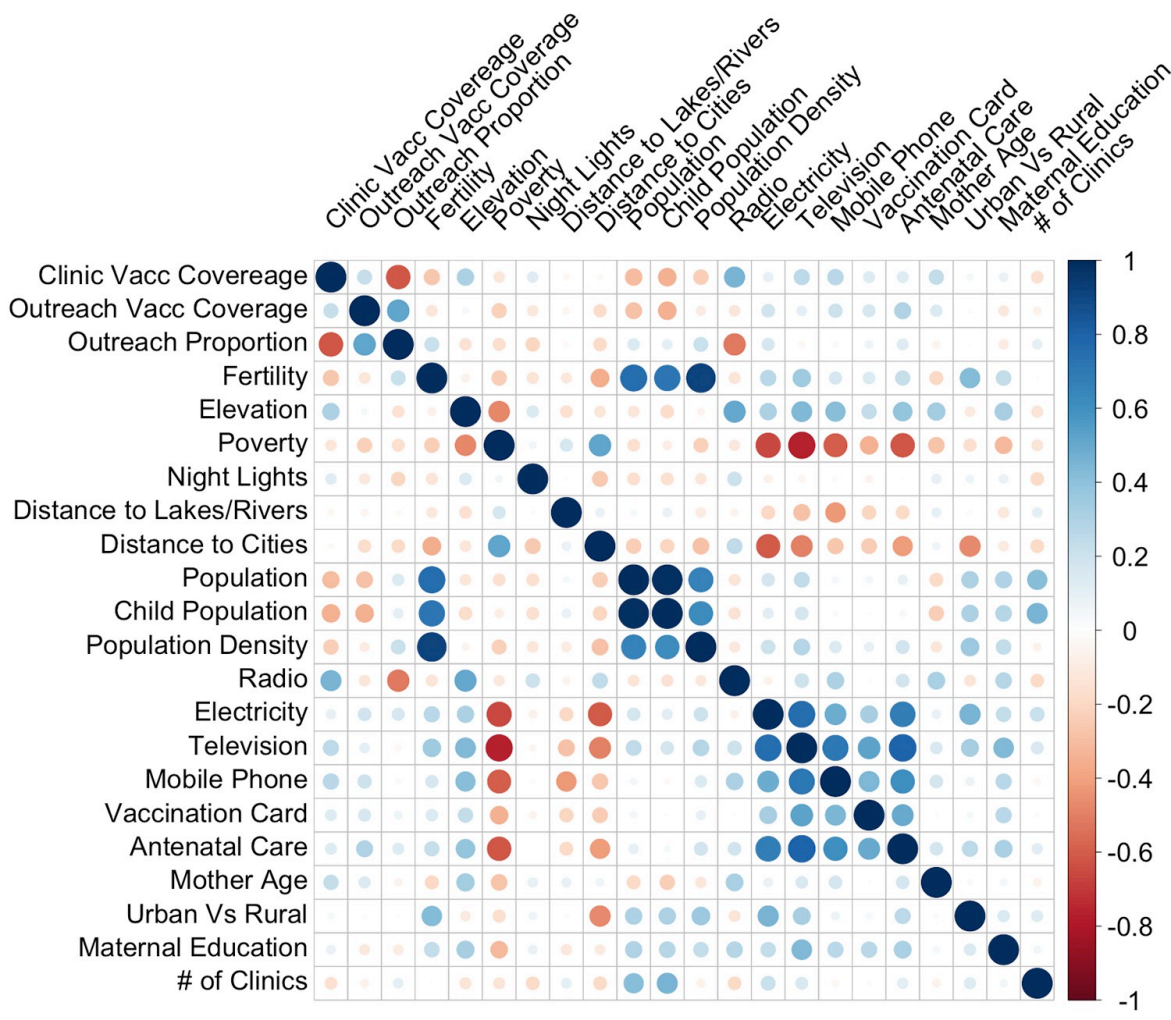
Correlation matrix at tehsil level. Correlation for all predictors and outcomes: outreach vaccinations, clinic vaccinations and the proportion of vaccinations that are outreach, by tehsil.

In terms of correlations, Fig 1 shows that outreach vaccinations and clinic vaccinations rates are both inversely correlated with the percentage of people living in poverty by region. These relations, once adjusted for all other regional attributes through multivariable analyses (ridge, GBM and GAM) are compiled for clinic vaccinations by tehsil, outreach vaccinations by tehsil and the proportion of outreach vaccinations by tehsil (Full results reported in S2 File) and are summarized below.

#### Predictors of clinic vaccination

**Fig 2:**
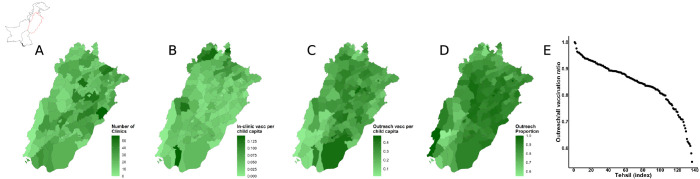
Clinics and vaccination distribution in Punjab province by tehsil. (A) Total number of clinics of each tehsil. (B) clinic vaccinations per 0–5-year child capita. (C) outreach vaccinations per child capita. (D) proportion of outreach out of all vaccinations. (E) scatter plot of proportion of outreach out of all vaccinations by tehsil. Base layer of the map was from the shape file provided by Humanitarian Data Exchange (source: Pakistan—Subnational Administrative Boundaries) [33].

Child population was in the top two relevant attributes in all three modelling methods. Though child population is important, more clinics are not in places where more penta-3 vaccinations occur (Fig 2A, 2B show the clinic distribution and clinic vaccinations per child capita by tehsil). Indeed, the relationship with child population in the ridge model showed a negative coefficient (GAM and GBM importance metrics do not show direction). Besides total population, other predictors with positive relationship with clinic vaccinations by tehsil (Ridge model) include: radio, television, night lights, and electricity, all related to electricity access (the opposite, lack of electricity consumption, has been correlated with poverty in Pakistan [32]). The same features were all significant in the GAM and GAB models as well. According to the ridge model results, the percentage of people living in poverty was positively related to in-clinic vaccination coverage controlling for other covariates, meaning places with increased poverty of a region had more penta-3 vaccinations happening (β = 0.077). Detailed tehsil attribute importance and ranking are described in S2 File.

#### Predictors of outreach vaccination

Fig 2C shows the outreach vaccinations per child capita distribution by tehsil. Child population and distance to cities were in the top five predictors for all models (negative relation). The predictors with positive relationship with outreach vaccinations by tehsil (ridge model) are: total population, % of mothers who sought antenatal care and mothers age, factors which are known to increase mothers’ productivity in child care [34]. The proportion of mothers who have sought antenatal care in the region was significant in all models, being the second most significant feature in the ridge model. Child population and average distance to cities by tehsil were common covariates with high significance in all outreach models and both with negative coefficients in the Ridge model. The proportion of households living in poverty was not significant in the outreach vaccination models. Detailed attribute importances are reported in S2 File.

#### Predictors of proportion outreach versus all vaccination

Tehsils with lower percentage of households with a radio was the most distinguishing predictor for a higher outreach vaccination proportion; meaning tehsils with a lower proportion of households with a radio is the most distinguishing factor, of all variables considered, of where there was more vaccination happening via outreach versus clinic vaccination. This could be the case as radio programs may broadcast public health information, such as where and when clinics are open for vaccination. Additionally, places with a higher percentage of mothers who sought antenatal care was the second significant attribute for a higher outreach vaccination proportion (positive relation) in the Ridge model, third in the GAM and fifth in the GBM. The proportion of people living in poverty in the region was the sixth most predictive feature in the ridge, eighth in the GAM and third in the GBM models and had a negative relation to outreach proportion (an increase in poverty corresponded to a decrease in the proportion of vaccinations happening by outreach). Population density, population and mean mother’s age in a region were positively related to outreach proportion. The range of outreach to clinic vaccination ratios by tehsil are illustrated in Fig 2E, and detailed results in S2 File.

#### Analyses at higher spatial resolution

Although not all attributes were available at the union council (fifth order administrative division) level, consistent results regarding a negative correlation between poverty and each of the clinic, outreach and outreach proportion were observed at this level (S1 Fig and S3 File). At the union council level, after controlling for other covariates, the percentage of people living in poverty was negatively related to outreach vaccination coverage and outreach vaccination proportion. Detailed model results are reported in S3 File.

## Discussion

### Outreach healthcare delivery

Our findings confirm that by the number of vaccinations occurring through outreach versus in clinics, outreach efforts are effective for reaching large populations beyond healthcare services through clinic facilities in Punjab, Pakistan. Further, the significant efforts that resulted in in digitizing healthcare delivery data through equipping all outreach and vaccinators are useful for analyzing the efforts, as they are leveraged here to understand how efforts are reaching populations based on regional attributes and thus how to plan healthcare delivery across multiple modalities (clinic and outreach).

### Area-based attribute importance and policy implications

As motivated above, regional attributes such as area-based demographics and healthcare resources can shape vaccination rates based on demand and access. In addition, area-based socioeconomic factors are also associated with a variety of health outcomes, independent of individual characteristics. Accordingly, based on analysis of regional attributes that were available at both spatial levels, there were consistent results regarding increased population density, and decreased poverty and elevation being related to outreach proportion at the tehsil and union council levels. Results show that outreach vaccinations are reaching places with more births and population (which are highly correlated, Fig 1), which is important. However, at the same time, conditional on population, places with increased poverty should also receive priority attention [4, 5, 7]. Overall, in multivariable analyses, factors related to childcare productivity and other assets (energy) show consistently significant, and positive relations with vaccination for both clinic and outreach vaccination. These results are important and immediately actionable. They show that outreach vaccinations in comparison to clinics are reaching more populated, less poor areas. Thus, accounting for this finding, outreach efforts can be adjusted. Without increasing resources for outreach vaccination, results indicate that vaccinations can be better targeted in order to reach populations who are facing barriers to vaccination. For instance, data on attendance at clinic locations cross-tabulated with other challenges such as poverty from different regions can be used in order to identify vulnerable groups who do not access clinics and have other barriers. These places can then be used to target outreach vaccinations specifically. Moreover, results of this work can also inform policy recommendations. For instance, World Health Organization guidelines currently recommend that community health programs adapt CHW catchments according to the local geography, including the proximity of households and population density. As results show, in order to ensure vulnerable populations are reached, added geographical factors such as area-based poverty or other barriers should also be a consideration for defining CHW catchments. Though more vaccinations were performed via outreach than in clinics, in multivariable analysis controlling for all included spatial attributes, outreach vaccination, based on the portable and mobile nature, especially with digitized recording tools, have opportunity to better reach those harder to reach (e.g. areas with higher poverty, higher elevation and/or higher distance to cities) [3].

### How do the predictors compare to results from other vaccination efforts?

In a previous study of vaccination coverage which included an examination of spatial attribute predictors for pentavalent vaccine from the Multiple Indicator Cluster Surveys (MICS) for Balochistan and Punjab (2010–2011) [9], access to a radio was associated with reduced pentavalent coverage. As our study only includes public system vaccinations, and outreach vaccinations outnumber clinic vaccinations, consistency with this previous study is not directly assessed (though the prior study is based on a limited number of data points garnered via survey and in a context where vaccinations were all delivered at clinics, while ours is based on millions of direct vaccination records including clinic and outreach efforts). Other studies of pentavalent vaccine have focused on identifying predictors of vaccination at larger spatial resolution and using those predictors to infer vaccination at higher spatial resolution. Despite the different focus of these studies and that vaccination efforts included in them are likely all from clinic efforts, predictors that were identified are comparable to findings in this study indicating consistent challenges with reaching marginal populations. For instance, a study across Africa found maternal education and income to be predictive of overall vaccination [28], while in the current study it was positively related to clinic vaccination rates and negative to outreach vaccination via correlation, maternal education was not included in multivariable analyses based on the feature selection methods. Another study in Pakistan on disaggregating DPT vaccination rates considered two covariates travel time to major cities of at least 50,000 people and population density, though neither were found significant in the final analysis [29].

### Limitations

There are several limitations to this study that must be considered when interpreting the results. First, this study only used data on public sector vaccination efforts. Since this is a very large and comprehensive dataset, and public sector care is free and thus more likely relevant for poor children which are of interest for this study, it was deemed a sufficient focus of examination. Second, variables at the individual level are not included due to data and privacy limitations. However, it is well known that spatial access inequities in vaccination persist and strategies that address access barriers in the hardest to reach communities at the regional level are needed for planning efforts and can be used to prioritize vulnerable populations [22, 35]. Finally, for the higher spatial-resolution analysis, not all predictors were available at the union council level and the performance of models at this level was low, suggesting that the accessible covariates are not sufficiently predictive of the outcome. However, since the results with respect to poverty were consistent with those from the tehsil-level analysis which included a wide range of relevant predictors, this gives indication that there are not large spatial heterogeneities in the relationship between poverty and outreach or clinic vaccination.

## Conclusion

This study was enabled by a large comprehensive digitized dataset which was a critical effort by the government of Punjab. This enabled examination of efforts in outreach vaccination over several years. While healthcare access through clinic facilities is critical and has been studied in depth [36], optimizing healthcare outreach services can fill important gaps in clinic-based care because of challenges including distance and cost. This was demonstrated by the large proportion of the population that received vaccination through outreach vaccination efforts, in comparison to accessing services at a clinic in Punjab. The literature has shown that in theory, CHWs can play an integral and dynamic role in overcoming community-specific barriers to expanding immunization coverage [3]. Our findings regarding the spatial attributes that predict current outreach vaccination efforts, are directly informative for moving this theory to action by informing specific studies and policies for CHW and resource allocation. Indeed, findings showing that spatial factors of increased population density, and decreased poverty and elevation are predictive of more outreach vaccination than clinic vaccinations, show that better strategy is needed to reach underserved populations. In addition to equipping health workers, in this case the mobile vaccination team, with the necessary tools to reach poor and hard-to-reach populations, planning of such efforts in complement to clinics and according to regional needs are critical next steps. There are persistent critical needs to reach vulnerable groups for vaccination and other healthcare services, and these needs also can potentially increase when healthcare institutions are less accessible during crises such as global pandemics [37]. Given these healthcare needs, along with an increased interest in technology-based efforts, our findings can be demonstrative for other national and sub-national regions facing challenges.

## Supporting information

S1 File Methods and results for assessing threshold for outreach data. (DOCX)

S2 File Details of predictors, model implementation, and model results at tehsil level. (DOCX)

S3 File Union council level analysis. (DOCX)

S4 File Model choice discussion and statistical checks. (DOCX)

S1 Table Location-based attributes, details, and sources. (DOCX)

S1 Fig Union council correlation results. Correlation matrix for all available predictors at the union council level, and three outcomes: outreach vaccinations, clinic vaccinations and the proportion of vaccinations that are outreach.
(TIF)
