# Supplementary material for: Area-based determinants of outreach vaccination for reaching vulnerable populations: A cross-sectional study in Pakistan
Source: PLOS Glob Public Health. 2023 Sep 27;3(9):e0001703. doi: 10.1371/journal.pgph.0001703 (PMC10529552; doi:10.1371/journal.pgph.0001703)

**Spatial determinants of outreach vaccination for reaching vulnerable populations in Pakistan - Methods and Results for Assessing Threshold for Outreach Data**

Precise GPS coordinates were provided with each vaccination record, though no field on whether the record occurred at a clinic or through outreach vaccination. Accordingly, in-clinic or outreach status of each vaccination record was determined based on distance (radius) from any clinic in the Punjab province. To determine the appropriate threshold for the distance, we first examined the ratio of clinic/outreach determination based on a range of possible thresholds (Supplementary Figure 1). We selected 0.0012$^{\circ}$ (~175 meters radius) as after this threshold, the proportion decreases linearly, and clustering before this could be due noise in the GPS coordinate values (known to exist up to 100m).

To assess robustness to this choice of the distance threshold we also reproduced all analyses in the paper using a 0.00045$^{\circ}$ buffer (~65 meters radius). It should be noted that all results were consistent. Major conclusions regarding poverty showed increased poverty was significantly associated with more in-clinic vaccinations (β =0.07 versus β =0.08 for 0.0012$^{\circ}$ threshold), and lower proportion of outreach vaccinations by region (β =-0.09 versus β = ­0.08 for 0.0012$^{\circ}$ threshold).

**Supplementary Figure A. Outreach proportion under different coordinates thresholds (red) computed using 5% (random) of all vaccination data.**


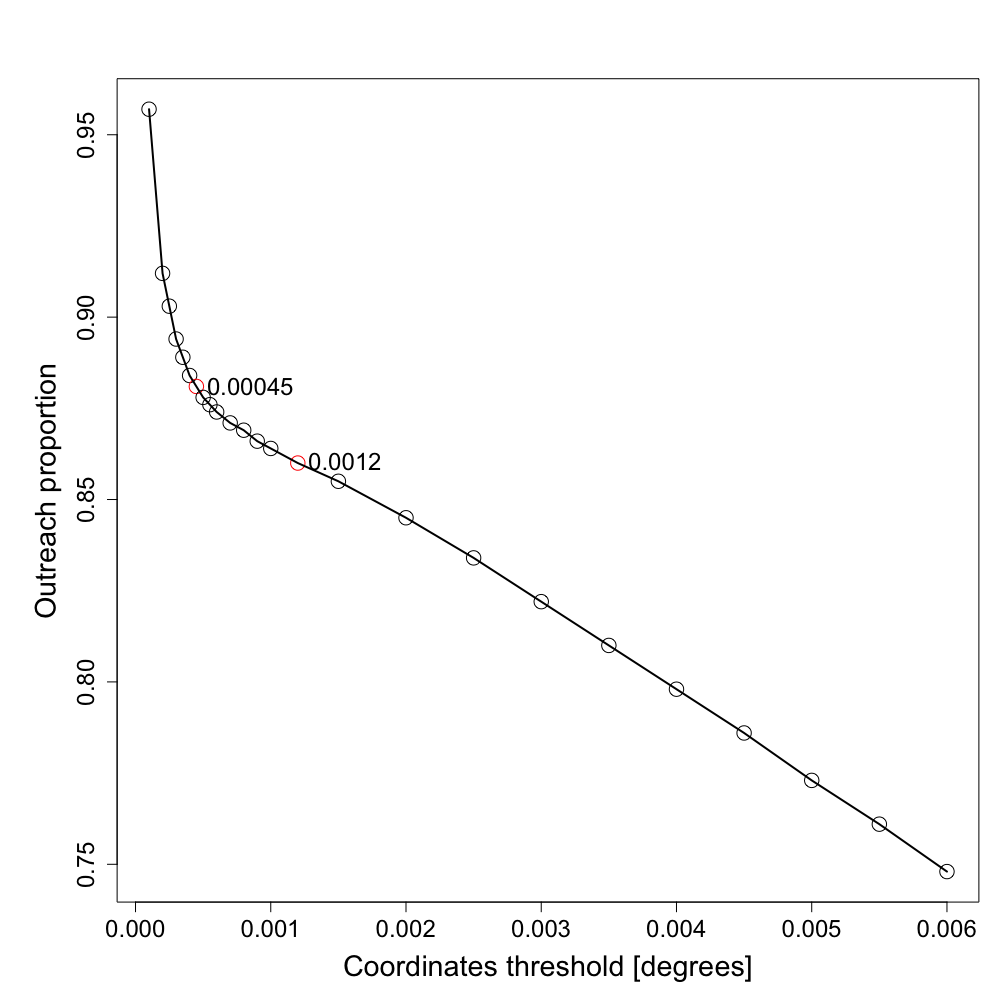

Supplement: S1 File — (DOCX) [file pgph.0001703.s001.docx]
