# Supplementary material for: Area-based determinants of outreach vaccination for reaching vulnerable populations: A cross-sectional study in Pakistan
Source: PLOS Glob Public Health. 2023 Sep 27;3(9):e0001703. doi: 10.1371/journal.pgph.0001703 (PMC10529552; doi:10.1371/journal.pgph.0001703)
Supplement: S2 File — (DOCX) [file pgph.0001703.s002.docx]

**Spatial determinants of outreach vaccination for reaching vulnerable populations in Pakistan – S2 File: Methods and Results for Understanding Relationship Between Spatial Attributes and Vaccination**

For the Tehsil level analysis, the feature selection process was as follows. For each GBM, GAM and ridge model, starting with all covariates recursive feature elimination was first performed using the Caret libraries Recursive Feature Elimination (RFE) Function (this simulates repeated 10 folds validation with 3 repeats to select which covariates are important). The Boruta function (via the Boruta package in R, with default random forest implementation) was also used to group each covariate as “confirmed”, “tentative” or “rejected” (outputs of the Boruta function) in terms of them being significant predictors for the given outcome. Covariates identified as important by both RFE and labelled as “confirmed” or “tentative” via Boruta were included in the ridge, GAM and GBM models.

Feature relevance were reported per best practices, and consistent with those reported previously in vaccination coverage prediction models [1]. For ridge regression, values of the standardized beta coefficients for the log-transformed covariates are given. For gradient boosted models (GBMs), relative influence was used for predictors. This indicates importance of the predictor in training the model. No direction is indicated. For generalized additive models (GAMs), the -log of the p-values for the smoothing function for a predictor (*s(∙)*) was used. Low p-values indicate low significance of smooth terms in the GAM.

For evaluation of all models, training and test were random 80/20 splits. The training set was standardized (z-score normalization) before model fitting, and the standardization of the test set was performed using the mean and the standard deviation of the training set. Overall model performance, as detailed below, are moderate. This is likely due to considering attributes at the area-level (no individual level attributes), however for the purposes of prediction and differentiation of vaccination types per region, are informative. Supplementary Table 4 presents model performance for each model in terms of the root mean squared error (RMSE), R-squared and Mean Absolute Error (MAE), all commonly used evaluation statistics [2]. While R-squared focuses on the goodness of fit, it can be inflated even when an insignificant predictor is added. On the other hand, RMSE and MAE serve a similar purpose in measuring the prediction ability of regression models. However, RMSE is more sensitive to outliers. In contrast, MAE has better interpretability since it is measured in the same units as the outcome variable. Each of the three metrics has its own merits, and when used together, they provide a more comprehensive evaluation of the fitted model. Further, RMSE performance level attained is comparable to that in other work assessing vaccine rates by region with respect to spatial predictors [3].

**Supplementary Table A. Clinic vaccination prediction resulting feature importance (tehsil level).**

| **GBM** | | | **GAM** | | | **Ridge** | | |
| --- | --- | --- | --- | --- | --- | --- | --- | --- |
|  | **Rel. influence** | **SE** |  | **­log**  **(p-value)** | **SE** |  | **Std Beta** | **SE** |
| radio | 15.71 | 2.24e-01 | child population | 3.31 | 8.36e-02 | (intercept) | 4.00e-16 | 2.18e-16 |
| child population | 13.68 | 2.38e-01 | radio | 3.27 | 6.72e-02 | log(child population) | -3.2e-01 | 1.11e-02 |
| mobile phone | 11.88 | 1.51e-01 | population | 2.86 | 7.06e-02 | log(radio) | 2.13e-01 | 2.42e-03 |
| poverty | 9.36 | 9.84e-02 | elevation | 2.23 | 7.06e-02 | log(television) | 1.83e-01 | 3.41e-03 |
| night lights | 7.35 | 7.15e-02 | poverty | 2.09 | 6.81e-02 | log(population) | 9.13e-02 | 1.07e-02 |
| mother age | 7.20 | 7.69e-02 | night lights | 1.87 | 6.39e-02 | log(pop density) | -8.80e-02 | 2.41e-03 |
| population | 6.79 | 7.00e-02 | electricity | 1.61 | 6.33e-02 | log(elevation) | 8.40e-02 | 3.81e-03 |
| elevation | 6.47 | 8.90e-02 | mother age | 1.40 | 5.18e-02 | log(poverty) | 7.68e-02 | 3.70e-03 |
| electricity | 6.34 | 6.23e-02 | mobile phone | 1.01 | 3.96e-02 | log(night lights) | 2.70e-02 | 3.15e-03 |
| fertility | 5.59 | 6.41e-02 | television | 0.93 | 3.94e-02 | log(mother age) | 1.71e-02 | 2.46e-03 |
| population density | 5.01 | 7.59e-02 | fertility | 0.81 | 3.28e-02 | log(mobile phone) | 1.57e-02 | 2.41e-03 |
| television | 4.63 | 5.33e-02 | population density | 0.72 | 2.89e-02 | log(fertility) | 6.18e-03 | 2.78e-03 |
|  |  |  |  |  |  | log(electricity) | 2.01e-03 | 3.51e-03 |

**Supplementary Table B. Outreach vaccination prediction resulting feature importance (tehsil level).**

| **GBM** | | | **GAM** | | | **Ridge** | | |
| --- | --- | --- | --- | --- | --- | --- | --- | --- |
|  | **Rel. influence** | **SE** |  | **­log**  **(p-value)** | **SE** |  | **Std Beta** | **SE** |
| mother age | 16.06 | 1.27e-01 | child population | 4.60 | 4.68e-02 | (intercept) | 5.78e-16 | 2.07e-16 |
| child population | 15.47 | 1.34e-01 | population | 3.13 | 4.08e-02 | log(child population) | -4.63e-01 | 6.46e-03 |
| elevation | 14.70 | 1.05e-01 | distance to cities | 2.50 | 4.81e-02 | log(antenatal care) | 2.77e-01 | 5.18e-03 |
| antenatal care | 14.48 | 1.15e-01 | night lights | 1.75 | 4.57e-02 | log(elevation) | -1.94e-01 | 4.27e-03 |
| distance to cities | 12.94 | 1.19e-01 | elevation | 1.40 | 3.75e-02 | log(distance to cities) | -1.66e-01 | 3.91e-03 |
| fertility | 9.15 | 7.36e-02 | mother age | 1.27 | 3.90e-02 | log(night lights) | -1.26e-01 | 3.79e-03 |
| night lights | 8.72 | 8.31e-02 | antenatal care | 1.13 | 3.50e-02 | log(fertility) | -1.13e-01 | 5.02e-03 |
| population | 8.47 | 6.76e-02 | fertility | 0.82 | 3.43e-02 | log(population) | 8.11e-02 | 5.65e-03 |
|  |  |  |  |  |  | log(mother age) | 5.74e-02 | 2.39e-03 |

**Supplementary Table C. Outreach proportion (outreach/all) vaccination prediction resulting feature importance (tehsil level).**

| **GBM** | | | **GAM** | | | **Ridge** | | |
| --- | --- | --- | --- | --- | --- | --- | --- | --- |
|  | **Rel. influence** | **SE** |  | **­log**  **(p-value)** | **SE** |  | **Std Beta** | **SE** |
| radio | 26.32 | 2.38e-01 | radio | 4.01 | 1.03e-01 | (intercept) | -2.80e-15 | 3.34e-16 |
| elevation | 9.65 | 1.08e-01 | electricity | 3.58 | 1.05e-01 | log(radio) | -3.96e-01 | 3.63e-03 |
| poverty | 8.97 | 1.03e-01 | antenatal care | 2.90 | 1.03e-01 | log(antenatal care) | 2.02e-01 | 3.73e-03 |
| night lights | 8.38 | 9.02e-02 | elevation | 2.53 | 1.15e-01 | log(fertility) | -1.58e-01 | 5.38e-03 |
| antenatal care | 8.16 | 8.08e-02 | child population | 2.13 | 1.15e-01 | log(elevation) | -1.26e-01 | 3.55e-03 |
| mobile phone | 7.23 | 7.11e-02 | population | 2.04 | 1.19e-01 | log(night lights) | -8.77e-02 | 2.85e-03 |
| mother age | 7.09 | 6.82e-02 | mother age | 1.84 | 7.58e-02 | log(poverty) | -8.31e-02 | 2.76e-03 |
| fertility | 5.83 | 5.63e-02 | poverty | 1.65 | 8.55e-02 | log(pop density) | 7.87e-02 | 5.06e-03 |
| electricity | 5.52 | 5.53e-02 | night lights | 1.65 | 7.84e-02 | log(child population) | -5.80e-02 | 6.03e-03 |
| child population | 4.65 | 5.24e-02 | mobile phone | 0.99 | 6.47e-02 | log(population) | 5.55e-02 | 5.61e-03 |
| population | 4.42 | 4.02e-02 | fertility | 0.77 | 5.28e-02 | log(electricity) | -1.97e-02 | 4.52e-03 |
| population density | 3.77 | 4.55e-02 | population density | 0.68 | 3.91e-02 | log(mother age) | 1.73e-02 | 2.59e-03 |
|  |  |  |  |  |  | log(mobile phone) | -7.89e-03 | 2.27e-03 |

**Supplementary Table D. Model performance for all Tehsil outcomes regarding RMSE, R-squared, and MAE**

|  | **RMSE** | | | **R-squared** | | | **MAE** | | |
| --- | --- | --- | --- | --- | --- | --- | --- | --- | --- |
|  | **GBM** | **GAM** | **RIDGE** | **GBM** | **GAM** | **RIDGE** | **GBM** | **GAM** | **RIDGE** |
| clinic coverage | 0.024 | 0.085 | 0.023 | 0.382 | 0.113 | 0.425 | 0.018 | 0.043 | 0.017 |
| outreach coverage | 0.083 | 0.106 | 0.077 | 0.149 | 0.092 | 0.201 | 0.070 | 0.079 | 0.058 |
| outreach proportion | 0.083 | 0.310 | 0.094 | 0.332 | 0.095 | 0.163 | 0.068 | 0.155 | 0.072 |

**References**

1. Mosser JF, Gagne-Maynard W, Rao PC, Osgood-Zimmerman A, Fullman N, Graetz N, et al. Mapping diphtheria-pertussis-tetanus vaccine coverage in Africa, 2000–2016: a spatial and temporal modelling study. The Lancet. 2019;393(10183):1843-55.
2. James, G., et al., *An introduction to statistical learning*. Vol. 112. 2013: Springer.
3. Utazi CE, Thorley J, Alegana V, Ferrari M, Nilsen K, Takahashi S, et al. A spatial regression model for the disaggregation of areal unit based data to high-resolution grids with application to vaccination coverage mapping. Statistical methods in medical research. 2019;28(10-11):3226-41.
