# Supplementary material for: Area-based determinants of outreach vaccination for reaching vulnerable populations: A cross-sectional study in Pakistan
Source: PLOS Glob Public Health. 2023 Sep 27;3(9):e0001703. doi: 10.1371/journal.pgph.0001703 (PMC10529552; doi:10.1371/journal.pgph.0001703)
Supplement: S3 File — (DOCX) [file pgph.0001703.s003.docx]

**Spatial determinants of outreach vaccination for reaching vulnerable populations in Pakistan – S3 File: Union Council Level Analysis**

For Union Council level analysis, the same feature selection procedure (RFE and Boruta) was used to select relative covariates from the 7 available spatial attributes, and the ridge model was implemented to determine feature relevance. Keeping in line with the tehsil level model, the same log transformation was applied for the UC ridge model. Bivariate linearity and homoscedastic were also checked for the ridge model. Since there is only a limited number of available features and 3 of them (*population*, *child population*, and *population density*) are highly correlated, the VIF criterion was used to choose the optimal lambda value for Ridge model fitting: choose the smallest lambda value while keeping all the VIF values no bigger than 10. The model performance is low (R-Squared close to or lower than 0.1) at the UC level, suggesting that the accessible covariates are not sufficiently predictive of the outcome, yet the feature relevance is supportive to the main tehsil level results.

**Supplementary Table E. Union council model feature importance (Ridge model) reported in order of absolute value.**

| **Clinic Model** | | | **Outreach Model** | | | **Outreach Proportion Model** | | |
| --- | --- | --- | --- | --- | --- | --- | --- | --- |
|  | **Std Beta** | **SE** |  | **Std Beta** | **SE** |  | **Std Beta** | **SE** |
| (intercept) | 1.7e-16 | 1.7e-17 | (intercept) | 1.3e-16 | 2.1e-17 | (intercept) | -1.4e-15 | 1.5e-16 |
| log(fertility) | -4.9e-02 | 1.5e-03 | log(pop density) | 4.8e-02 | 1.3e-06 | log(pop density) | 4.7e-02 | 1.2e-06 |
| log(poverty) | -3.5e-02 | 10.0e-04 | log(poverty) | -4.0e-03 | 4.8e-05 | log(population) | -8.1e-03 | 3.3e-05 |
| log(distance to cities) | -2.0e-02 | 9.6e-04 | log(child population) | 3.6e-03 | 1.3e-04 | log(distance to cities) | -7.7e-03 | 3.3e-05 |
|  |  |  | log(population) | -2.9e-03 | 1.3e-04 | log(poverty) | -4.0e-03 | 2.9e-05 |
|  |  |  | log(elevation) | 1.1e-03 | 1.5e-05 | log(fertility) | 3.2e-03 | 2.6e-05 |
|  |  |  |  |  |  | log(child population) | 2.6e-03 | 2.6e-05 |
|  |  |  |  |  |  | log(elevation) | -1.0e-03 | 3.4e-05 |
