# Supplementary material for: Area-based determinants of outreach vaccination for reaching vulnerable populations: A cross-sectional study in Pakistan
Source: PLOS Glob Public Health. 2023 Sep 27;3(9):e0001703. doi: 10.1371/journal.pgph.0001703 (PMC10529552; doi:10.1371/journal.pgph.0001703)
Supplement: S4 File — (DOCX) [file pgph.0001703.s004.docx]

**Spatial determinants of outreach vaccination for reaching vulnerable populations in Pakistan – S4 File: Model Choice Discussion and Statistical Checks**

Reasoning for model choices:

Ideal models for this task should be flexible enough to handle complex relationships between spatial factors and area-based vaccination rates. At the same time, interpretability is important to understand the contribution and direction of association of each possible spatial factor. Thus in this work we select three common modelling approaches in order to retain the benefits of each: i) Gradient Boosting Machines (GBMs) which can capture nonlinear relationships and works well for prediction even with multicollinearity and heteroskedasticity issues without any special treatments, though lack in intepretability, ii) Generalized Additive Models (GAMs) which allow for nonlinear relationships though still retain interpretability and iii) Ridge regression which are the least flexible of the three, yet provide clear interpretable coefficients for interpretation of the magnitude and direction of the predictors. Given tradeoffs with each modelling approach, including results from all three models allows comparison of results for interpretation of results.

Statistical checks:

Ridge is a regularized extension of linear regression; hence linearity and homoscedasticity are assumed. The normality of residual distribution is not assumed since ridge regression does not provide confidence limits. The multicollinearity issue can also be addressed through the nature of regularization, which can effectively shrink the coefficient estimates of correlated variables. Given that the relationship between most covariates and the outcome is not linear, log-transformation was applied to the independent variables to meet the assumption of linearity. The residual plots of models suggest that the assumption of homoscedasticity is also satisfied after log transformation.

For GAM models, is it important to check the residual plots like many generalized linear models, and the degree of freedom of each smooth term to make sure the choice of basis dimension was not restrictively small. The “gam.check” function from “mgcv” package in R helps with the diagnostics of fitted GAM models. Upon checking on all three GAM models, the assumption for normality and homoscedasticity of residuals generally holds, and the diagnostics regarding the degree of freedom do not reveal significant problems.
