# Supplementary material for: Area-based determinants of outreach vaccination for reaching vulnerable populations: A cross-sectional study in Pakistan
Source: PLOS Glob Public Health. 2023 Sep 27;3(9):e0001703. doi: 10.1371/journal.pgph.0001703 (PMC10529552; doi:10.1371/journal.pgph.0001703)
Supplement: S1 Table — (DOCX) [file pgph.0001703.s005.docx]

**Spatial determinants of outreach vaccination for reaching vulnerable populations in Pakistan – Variable information**

**S1 Table: Location-based attributes, details, and sources.**

| **Feature** | **Description (per region)** | **Source** | **Details** | **# missing values** | **Available at UC level** | **Reasoning for inclusion** |
| --- | --- | --- | --- | --- | --- | --- |
| Child Population | Child under 5 population | Facebook/Columbia high resolution population data (1) |  | 0 | Yes | Demographic features of the region provide valuable information about the target population. They are the basis of understanding the healthcare demand, resource allocation, and accessibility of the area,  thus, may influence the vaccination coverage rates. As reference, population, population density, and fertility have been shown to have relevance in the prediction of vaccination coverage (2-5). |
| Population | Total population | Facebook/Columbia high resolution population data (1) |  | 0 | Yes |  |
| Population Density | Total population / area of region | Facebook/Columbia high resolution population data (1) |  | 0 | Yes |  |
| Fertility | Total births in region / population | WorldPop (6) | Extracted from TIF File | 0 | Yes |  |
| Night Lights | Geographic lights present at night approximation from satellite imagery in region (nightlight development index) | NOAA­ National Oceanic and  Atmospheric Administration Documentation (7) | Extracted from TIF file | 12 |  | These are the socioeconomics indicators of the regions. Low socioeconomic status has been shown to be related to low vaccination rates in developing countries (8, 9) and it has been significantly associated with influenza vaccination in developed countries in Europe (10). Further, concentrated socioeconomic disadvantage may also diminish institutional resources through which information and social support, including that related to obtaining health care may be obtained, thus influencing vaccination rates (11). |
| Electricity | % of households with electricity | MICS Survey 2011 (12) |  | 0 |  |  |
| Poverty | % of people living in poverty | WorldPop (6) | Extracted from TIF File | 0 | Yes |  |
| Radio | % of households with a radio | Multiple Indicator Cluster Survey  (MICS) Survey 2011 (12) |  | 0 |  | Media exposure reflect information access, which can significantly influence both awareness of importance of receiving vaccination and understanding access to vaccination resources. The latter is especially important in this study, where we are interested specifically in outreach vaccination coverage. For outreach vaccination, as there are no fixed locations like traditional clinics, people's awareness of it relies heavily on media dissemination and word of mouth among individuals. Poor media access has also been shown to be related to low vaccination rates (8). |
| Mobile Phone | % of females with a mobile phone | MICS Survey 2011 (12) |  | 0 |  |  |
| Television | % of households with a television | MICS Survey 2011 (12) |  | 0 |  |  |
| Antenatal Care | % of mothers who sought antenatal care | MICS 2011 Survey (12) |  | 0 |  | These covariates reflect the characteristics of the mother population in the area based on the important role mothers play in the immunization process. Mothers’ age and education are related to vaccination awareness and uptake. Previous studies have shown associations between children’s vaccination and mother’s age and education level (9, 13, 14). Exposure to antenatal care indicates awareness and access to medical resources, the association between antenatal care visits and vaccine completion has also been revealed (9). Therefore, these factors are likely to influence vaccination coverage rates. |
| Mothers Age | Average age of mothers (years) | MICS Survey 2011 (12) |  | 0 |  |  |
| Maternal Education | Average level of mother’s education | MICS 2011 Survey (12) | Coded in 5 categories: ‘preschool’ = 1; ‘primary’ = 2; ‘middle’ = 3; ‘matric’ =4, ‘above matric’ = 5. Mode used to represent each Tehsil. | 0 |  |  |
| Elevation | Average geographic elevation (meters) | DIVA­GIS (15) | Extracted from GRD  File | 0 | Yes | Elevation, urban-rural ratio, distance to cities, and distance to lakes/rivers are geographical features that can directly affect the development of healthcare facilities and people’s accessibility to healthcare resources. Populations under disadvantaged geographic conditions are likely to suffer from lower vaccine coverage rates (8). Research has also shown that the urban rural ratio is a determinant of children immunization (14). Vaccination card and the number of clinics are also indicators of accessibility to vaccination resources. Populations located near health facilities are more likely to have a higher vaccination coverage rates (16-18). |
| Urban Rural Ratio | Ratio of people living in urban to rural areas | MICS 2011 Survey (12) |  | 0 |  |  |
| Distance to Cities | Average distance to a major city from all points in region (km) | WorldPop (6) |  | 0 | Yes |  |
| Vaccination Card | % of people who have a vaccina­  tion card | MICS 2011 Survey (12) |  | 0 |  |  |
| Number of clinics | Total number of public vaccination clinics in region | Study data |  | 0 |  |  |
| Distance to Lakes/Rivers | Average distance to lakes/rivers from all points in region | Natural Earth: Rivers and lake centerlines dataset (19) |  | 0 |  |  |

**References**

1. Facebook Connectivity Lab and Center for International Earth Science Information Network – CIESIN – Columbia University. High Resolution Settlement Layer (HRSL).

2. Mosser JF, Gagne-Maynard W, Rao PC, Osgood-Zimmerman A, Fullman N, Graetz N, et al. Mapping diphtheria-pertussis-tetanus vaccine coverage in Africa, 2000–2016: a spatial and temporal modelling study. The Lancet. 2019;393(10183):1843-55.

3. Utazi CE, Thorley J, Alegana VA, Ferrari MJ, Takahashi S, Metcalf CJE, et al. High resolution age-structured mapping of childhood vaccination coverage in low and middle income countries. Vaccine. 2018;36(12):1583-91.

4. Utazi CE, Thorley J, Alegana VA, Ferrari MJ, Takahashi S, Metcalf CJE, et al. Mapping vaccination coverage to explore the effects of delivery mechanisms and inform vaccination strategies. Nature communications. 2019;10(1):1633.

5. Mapping routine measles vaccination in low-and middle-income countries. Nature. 2021;589(7842):415-9.

6. Lloyd CT, Sorichetta A, Tatem AJ. High resolution global gridded data for use in population studies. Scientific data. 2017;4(1):1-17.

7. National Centers for Environmental Information. Night Light Development Index (NLDI). 2006.

8. Rainey JJ, Watkins M, Ryman TK, Sandhu P, Bo A, Banerjee K. Reasons related to non-vaccination and under-vaccination of children in low and middle income countries: findings from a systematic review of the published literature, 1999–2009. Vaccine. 2011;29(46):8215-21.

9. Nozaki I, Hachiya M, Kitamura T. Factors influencing basic vaccination coverage in Myanmar: secondary analysis of 2015 Myanmar demographic and health survey data. BMC public health. 2019;19(1):1-8.

10. Endrich MM, Blank PR, Szucs TD. Influenza vaccination uptake and socioeconomic determinants in 11 European countries. Vaccine. 2009;27(30):4018-24.

11. Browning CR, Cagney KA. Neighborhood structural disadvantage, collective efficacy, and self-rated physical health in an urban setting. Journal of health and social behavior. 2002:383-99.

12. United Nations Children’s Fund. Multiple Indicator Cluster Surveys. 2011.

13. Ibnouf A, Van den Borne H, Maarse J. Factors influencing immunisation coverage among children under five years of age in Khartoum State, Sudan. South African Family Practice. 2007;49(8):14-f.

14. Munthali AC. Determinants of vaccination coverage in Malawi: evidence from the demographic and health surveys. Malawi Medical Journal. 2007;19(2):79-82.

15. Hijmans RJ, Guarino L, Cruz M, Rojas E. Computer tools for spatial analysis of plant genetic resources data: 1. DIVA-GIS. Plant Genetic Resources Newsletter. 2001:15-9.

16. Otieno NA, Nyawanda BO, Audi A, Emukule G, Lebo E, Bigogo G, et al. Demographic, socio-economic and geographic determinants of seasonal influenza vaccine uptake in rural western Kenya, 2011. Vaccine. 2014;32(49):6699-704.

17. Sanou A, Simboro S, Kouyaté B, Dugas M, Graham J, Bibeau G. Assessment of factors associated with complete immunization coverage in children aged 12-23 months: a cross-sectional study in Nouna district, Burkina Faso. BMC international health and human rights. 2009;9:1-15.

18. Nanthavong N, Black AP, Nouanthong P, Souvannaso C, Vilivong K, Muller CP, et al. Diphtheria in Lao PDR: insufficient coverage or ineffective vaccine? PloS one. 2015;10(4):e0121749.

19. Natural Earth. Natural Earth. Rivers and lake centerlines dataset. 2018 March 2, 2020.
