# Supplementary figures and images for: Area-based determinants of outreach vaccination for reaching vulnerable populations: A cross-sectional study in Pakistan
Source: PLOS Glob Public Health. 2023 Sep 27;3(9):e0001703. doi: 10.1371/journal.pgph.0001703 (PMC10529552; doi:10.1371/journal.pgph.0001703)

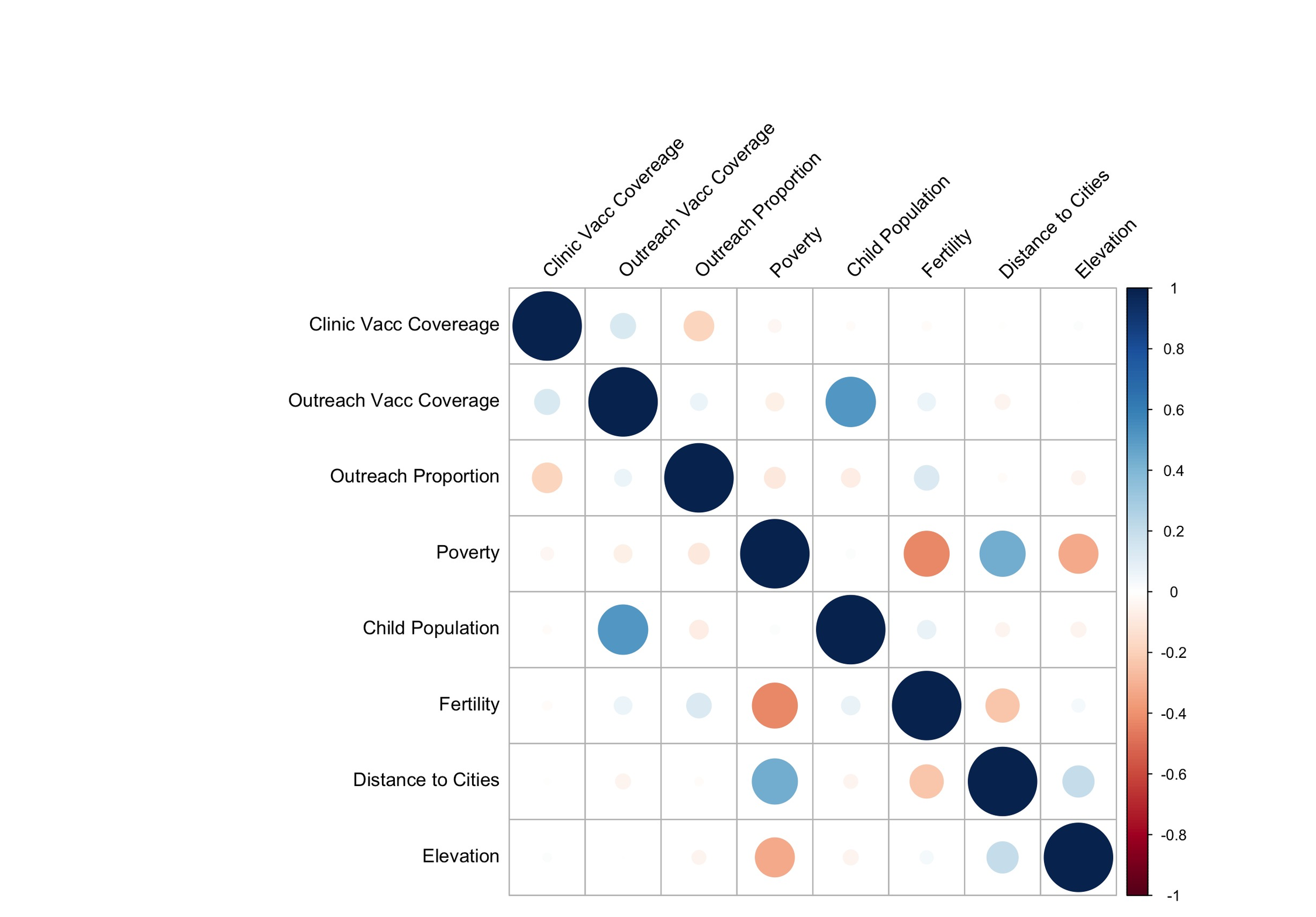

Supplement: S1 Fig — Correlation matrix for all available predictors at the union council level, and three outcomes: outreach vaccinations, clinic vaccinations and the proportion of vaccinations that are outreach. (TIF) [file pgph.0001703.s006.tif]
